# Draft Genome Sequence of Bacillus subtilis YBS29, a Potential Fish Probiotic That Prevents Motile *Aeromonas* Septicemia in Labeo rohita

**DOI:** 10.1128/mra.00915-22

**Published:** 2022-09-26

**Authors:** Sulav Indra Paul, M. Mahbubur Rahman

**Affiliations:** a Institute of Biotechnology and Genetic Engineering, Bangabandhu Sheikh Mujibur Rahman Agricultural University, Gazipur, Bangladesh; University of Rochester School of Medicine and Dentistry

## Abstract

We report the draft genome sequence of the promising fish probiotic Bacillus subtilis YBS29. This strain exhibits *in vitro* antimicrobial activity against Aeromonas veronii and enhances growth and disease resistance in the Indian major carp species Labeo rohita against motile *Aeromonas* septicemia (MAS). Its genome contains a gene cluster encoding multiple bacteriocins and lacks genes for virulence factors. These genomic features signify potential for safe use as a probiotic in aquaculture.

## ANNOUNCEMENT

Bacillus subtilis YBS29 was isolated from a marine sponge species (Hemimycale columella) and identified as described previously by Paul et al. (1). It exhibits *in vitro* inhibitory activity against pathogenic Aeromonas veronii, prevents MAS in Labeo rohita, and is considered a promising fish probiotic candidate (1). Prior permission was received from the IBGE ethical review committee for the animal experiments (approval number IBGE-ERC-008).

High-quality genomic DNA of B. subtilis YBS29 was extracted from an overnight culture in Zobell broth at 28°C using a GeneJET genomic DNA purification kit (Thermo Fisher Scientific, USA), and the quality and quantity of the DNA were checked using a NanoDrop spectrophotometer (Thermo Fisher Scientific). The bacterium was identified as B. subtilis (GenBank accession number MT605348.1) based on 16S rRNA gene sequence homology, as described previously (1). A DNA library was prepared using the Nextera XT library prep kit (Illumina, San Diego, CA, USA) according to the manufacturer’s instructions (2). Sequencing (600 cycles) was performed using the MiSeq benchtop sequencer (Illumina, Inc.) (3, 4) and yielded 2,392,312 paired-end reads. The Bacterial Analysis Pipeline SpeciesFinder v2.0 was used for the initial identification of the bacterium (5). Sequence adaptors were removed using Trimmomatic v0.38 (6), and quality filtering was conducted using PRINSEQ v0.20.3 (7). *De novo* assembly was performed using SPAdes v3.9.0 (8), and quality evaluation of the assembled genome sequence was carried out using QUAST v5.0.2 (9). Genome annotation was performed using the NCBI Prokaryotic Genome Annotation Pipeline (PGAP) (10). Default parameters were used for all software unless otherwise specified.

The *de novo* assembly resulted in an estimated chromosome size of 4,064,081 bp (49 contigs), with 43.6% G+C content from 2,392,312 paired-end reads and a total of 986,379,456 bases sequenced, providing 243× coverage. The genome contains 4,272 coding sequences and 106 RNA genes as predicted using PGAP (81 tRNA, 20 rRNA, and 5 noncoding RNA [ncRNA] genes). RAST analysis (11) predicted 332 subsystems and 1,692 protein-coding genes in putative functional categories. The *N*_50_ and *L*_50_ values of the assembly were 153,460 bp and 7, respectively. The largest and smallest contigs were 438,409 bp and 547 bp, respectively. No remarkable antibiotic-resistant genes, no genes encoding putative virulence factors, and no plasmids were identified in the genome using ResFinder v4.1 (12), VirulenceFinder v2.0 (13), and PlasmidFinder v2.1 (14), respectively, with default parameters. Using antiSMASH v5.1.2, the genome was determined to encode several orthologs of intrinsic genes of antimicrobial peptides, including surfactin, subtilin, bacillibactin, bacilysin, subtilosin A, bacillaene, fengycin, and plipastatin (15). The genome encodes bacitracin stress response genes (*BceA*, *BceB*, *BceR*, and *BceS*) and denitrifying reductase genes (*NorD* and *NorQ*). Additionally, bacteriocin synthesis gene clusters coding for salivaricin D, flavucin, entianin, ericin A and S, mejucin, nisin (A, F, Q, U, and Z), and subtilosin (*SboX*) were detected in the genome using BAGEL4 (16). The presented genome information will assist further studies of this strain to exploit its probiotic potential.

### Data availability.

The whole-genome shotgun project of B. subtilis strain YBS29 has been deposited at GenBank under the accession number JANTOL000000000. The raw sequence reads are available under the SRA accession number SRX15796884, BioProject accession number PRJNA796512, and the BioSample accession number SAMN24891510.

## References

[1] Paul SI, Rahman MM, Salam MA, Khan MA, Islam MT. 2021. Identification of marine sponge-associated bacteria of the Saint Martin's Island of the Bay of Bengal emphasizing on the prevention of motile *Aeromonas* septicemia in *Labeo rohita*. Aquaculture 545:737156. doi:10.1016/j.aquaculture.2021.737156.

[2] Illumina. 2019. Nextera XT DNA library prep: reference guide. Illumina, San Diego, CA. https://support.illumina.com/content/dam/illumina-support/documents/documentation/chemistry_documentation/samplepreps_nextera/nextera-xt/nextera-xt-library-prep-reference-guide-15031942-05.pdf.

[3] Rahman MM, Paul SI, Akter T, Tay AC, Foysal MJ, Islam MT. 2020. Whole-genome sequence of *Bacillus subtilis* WS1A, a promising fish probiotic strain isolated from marine sponge of the Bay of Bengal. Microbiol Resour Announc 9:e00641-20. doi:10.1128/MRA.00641-20. 32972930 PMC7516141

[4] Akter T, Rahman MM, Tay ACY, Ehsan R, Islam MT. 2020. Whole-genome sequence of fish-pathogenic *Enterococcus faecalis* strain BFFF11. Microbiol Resour Announc 9:e01447-19. doi:10.1128/MRA.01447-19. 32054709 PMC7019064

[5] Larsen MV, Cosentino S, Lukjancenko O, Saputra D, Rasmussen S, Hasman H, Sicheritz-Pontén T, Aarestrup FM, Ussery DW, Lund O. 2014. Benchmarking of methods for genomic taxonomy. J Clin Microbiol 52:1529–1539. doi:10.1128/JCM.02981-13. 24574292 PMC3993634

[6] Bolger AM, Lohse M, Usadel B. 2014. Trimmomatic: a flexible trimmer for Illumina sequence data. Bioinformatics 30:2114–2120. doi:10.1093/bioinformatics/btu170. 24695404 PMC4103590

[7] Schmieder R, Edwards R. 2011. Quality control and preprocessing of metagenomic datasets. Bioinformatics 27:863–864. doi:10.1093/bioinformatics/btr026. 21278185 PMC3051327

[8] Bankevich A, Nurk S, Antipov D, Gurevich AA, Dvorkin M, Kulikov AS, Lesin VM, Nikolenko SI, Pham S, Prjibelski AD, Pyshkin AV, Sirotkin AV, Vyahhi N, Tesler G, Alekseyev MA, Pevzner PA. 2012. SPAdes: a new genome assembly algorithm and its applications to single-cell sequencing. J Comput Biol 19:455–477. doi:10.1089/cmb.2012.0021. 22506599 PMC3342519

[9] Gurevich A, Saveliev V, Vyahhi N, Tesler G. 2013. QUAST: quality assessment tool for genome assemblies. Bioinformatics 29:1072–1075. doi:10.1093/bioinformatics/btt086. 23422339 PMC3624806

[10] Tatusova T, DiCuccio M, Badretdin A, Chetvernin V, Nawrocki EP, Zaslavsky L, Lomsadze A, Pruitt KD, Borodovsky M, Ostell J. 2016. NCBI Prokaryotic Genome Annotation Pipeline. Nucleic Acids Res 44:6614–6624. doi:10.1093/nar/gkw569. 27342282 PMC5001611

[11] Overbeek R, Olson R, Push GD, Olsen GJ, Davis JJ, Disz T, Edwards RA, Gerdes S, Parrello B, Shukla M, Vonstein V, Wattam AR, Xia F, Stevens R. 2014. The SEED and the Rapid Annotation of microbial genomes using Subsystems Technology (RAST). Nucleic Acids Res 42:D204–D214. doi:10.1093/nar/gkt1226. PMC3965101 24293654

[12] Zankari E, Hasman H, Cosentino S, Vestergaard M, Rasmussen S, Lund O, Aarestrup FM, Larsen MV. 2012. Identification of acquired antimicrobial resistance genes. J Antimicrob Chemother 67:2640–2644. doi:10.1093/jac/dks261. 22782487 PMC3468078

[13] Joensen KG, Scheutz F, Lund O, Hasman H, Kaas RS, Nielsen EM, Aarestrup FM. 2014. Real-time whole-genome sequencing for routine typing, surveillance, and outbreak detection of verotoxigenic *Escherichia coli*. J Clin Microbiol 52:1501–1510. doi:10.1128/JCM.03617-13. 24574290 PMC3993690

[14] Carattoli A, Zankari E, García-Fernández A, Larsen MV, Lund O, Villa L, Aarestrup FM, Hasman H. 2014. *In silico* detection and typing of plasmids using PlasmidFinder and plasmid multilocus sequence typing. Antimicrob Agents Chemother 58:3895–3903. doi:10.1128/AAC.02412-14. 24777092 PMC4068535

[15] Blin K, Wolf T, Chevrette MG, Lu X, Schwalen CJ, Kautsar SA, Suarez Duran HG, de los Santos ELC, Kim HU, Nave M, Dickschat JS, Mitchell DA, Shelest E, Breitling R, Takano E, Lee SY, Weber T, Medema MH. 2017. antiSMASH 4.0—improvements in chemistry prediction and gene cluster boundary identification. Nucleic Acids Res 45:W36–W41. doi:10.1093/nar/gkx319. 28460038 PMC5570095

[16] van Heel AJ, de Jong A, Song C, Viel JH, Kok J, Kuipers OP. 2018. BAGEL4: a user-friendly Web server to thoroughly mine RiPPs and bacteriocins. Nucleic Acids Res 46:W278–W281. doi:10.1093/nar/gky383. 29788290 PMC6030817

